# Supplementary material for: Separating mitochondrial protein assembly and endoplasmic reticulum tethering by selective coupling of Mdm10
Source: Nat Commun. 2016 Oct 10;7:13021. doi: 10.1038/ncomms13021 (PMC5476798; doi:10.1038/ncomms13021)
Supplement: Supplementary Information — Supplementary Figures 1-6, Supplementary Table 1 and Supplementary References. [file ncomms13021-s1.pdf]

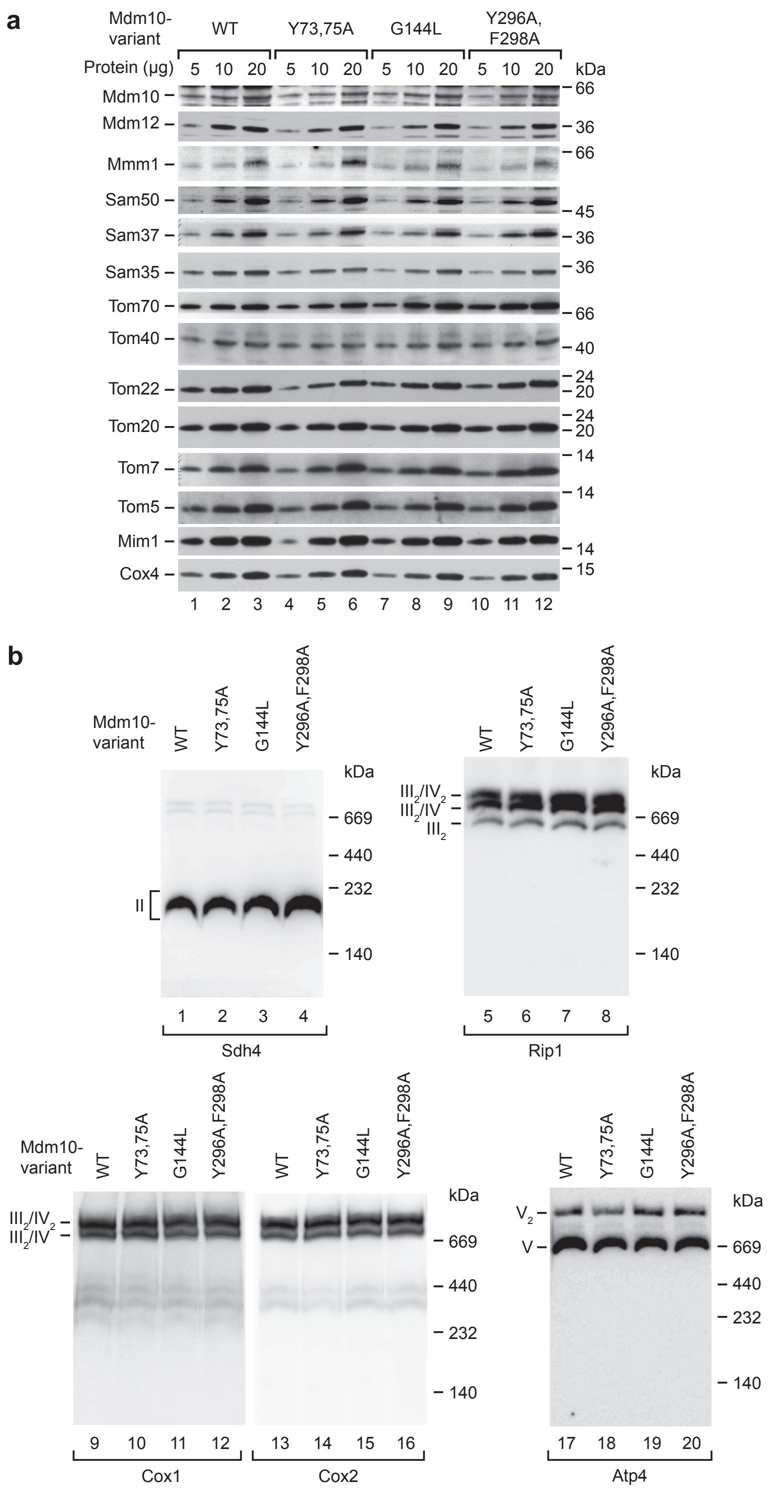

Supplementary Figure 1 | Steady-state protein levels of *mdm10* mutant mitochondria. (**a**) Mitochondria isolated from cells expressing wild-type (WT) or mutant forms of *MDM10* were lysed with SDS. The indicated amounts of mitochondrial proteins were separated by SDS-PAGE. Mitochondrial proteins were detected by immunodecoration with the indicated antisera. Mim1, mitochondrial import protein 1; Cox4, cytochrome *c* oxidase subunit 4. (**b**) Mitochondria isolated from cells expressing WT or mutant forms of *MDM10* were solubilized with digitonin. Oxidative phosphorylation complexes were analyzed by blue native electrophoresis and immunodetection with the indicated antisera. II, complex II (succinate dehydrogenase); III, complex III (cytochrome *c* reductase); IV; complex IV (cytochrome *c* oxidase); V, complex V (F_1F_o-ATP synthase) of mitochondrial oxidative phosphorylation machinery. Rip1, Rieske iron-sulfur protein.

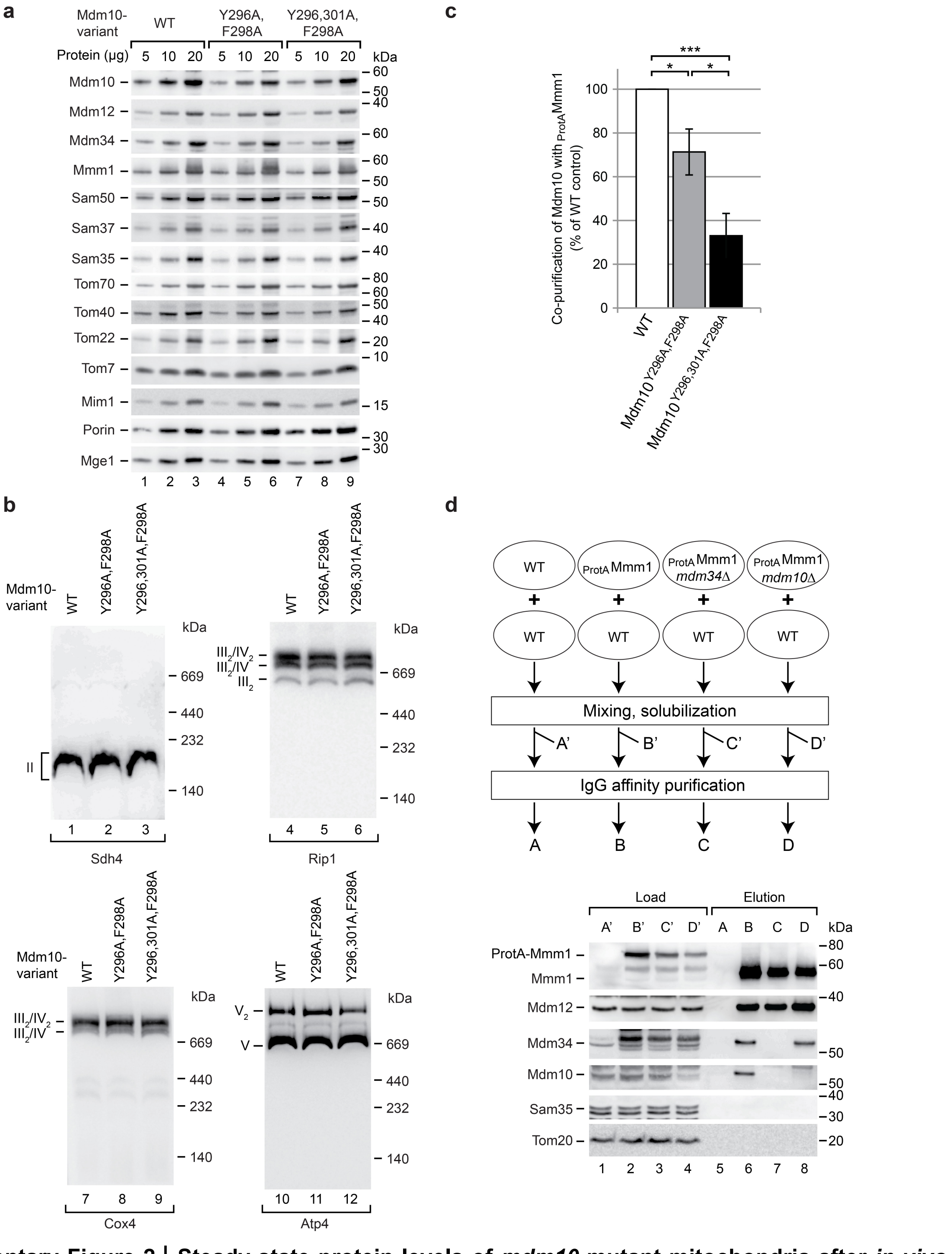

Supplementary Figure 2 | Steady-state protein levels of *mdm10* mutant mitochondria after *in vivo* heat shock and quantification of co-purification. (**a**) Wild-type (WT), Mdm10^{Y296A,F298A} and Mdm10^{Y296,301A,F298A} cells were grown in YPG medium at 30 °C to mid-log phase and shifted to 37 °C for 3 h. Mitochondria were isolated and lysed with SDS. The indicated amounts of proteins were separated by SDS-PAGE and analyzed by immunodecoration with the indicated antisera. Mge1, mitochondrial GrpE. (**b**) WT, Mdm10^{Y296A,F298A} and Mdm10^{Y296,301A,F298A} cells were grown in YPG medium at 30 °C to mid-log phase and shifted to 37 °C for 3 h. Mitochondria were isolated and solubilized with digitonin. Oxidative phosphorylation complexes were analyzed by blue native electrophoresis and immunodetection with the indicated antisera. (**c**) Quantification of the co-purification of Mdm10 with $_{ProtA}$Mmm1 (performed as described in Fig. 2c). The yield of co-purification in WT was set to 100% (control). Data are presented as mean ± s.e.m. (n = 4). Statistically significant differences are indicated by asterisks (unpaired *t-test* with *$p < 0.05$ and ***$p < 0.001$). (**d**) Whole cell extracts from WT, $_{ProtA}$Mmm1, $_{ProtA}$Mmm1 *mdm34*Δ and $_{ProtA}$Mmm1 *mdm10*Δ cells were mixed with equal amounts of whole cell extracts from WT cells, solubilized with digitonin and subjected to affinity purification via IgG-Sepharose. Load 5%, elution 100%.

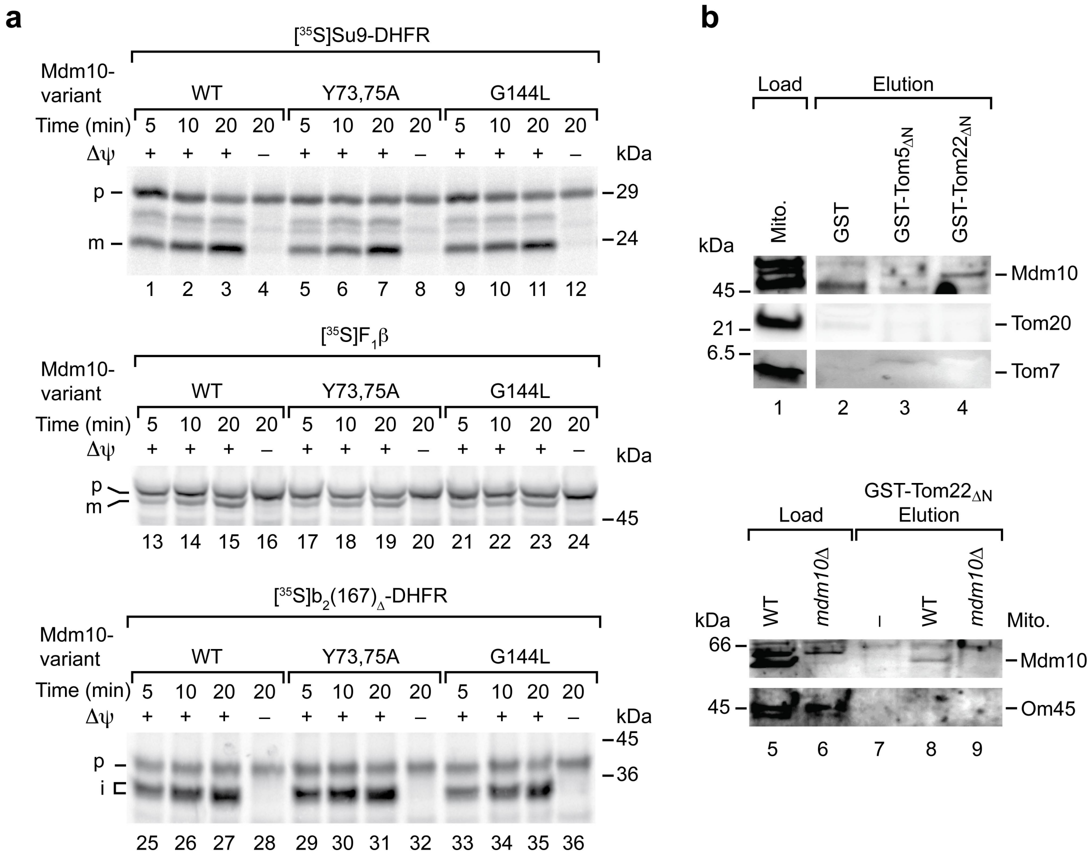

Supplementary Figure 3 | Import of cleavable preproteins into *mdm10* mutant mitochondria and binding of Mdm10 to Tom22. (**a**) The presequence-carrying preproteins [35S]Su9-DHFR, [35S]F$_1$β and [35S]b$_2$(167)$_\Delta$-DHFR were imported into mitochondria isolated from cells expressing wild-type (WT) or mutant forms of *MDM10.* Mitochondria were analyzed by SDS-PAGE and autoradiography. Δψ, membrane potential; p, precursor; i, intermediate; m, mature. (**b**) Upper panel, WT mitochondria were solubilized with digitonin and incubated with affinity matrix coated with GST, GST-Tom5$_{\Delta N}$ (Tom5 amino acid residues 16-50) or GST-Tom22$_{\Delta N}$ (Tom22 amino acid residues 85-152). The proteins fused to GST were eluted and the samples were analyzed by SDS-PAGE. Load 19%, elution 100%. Lower panel, mitochondria isolated from *mdm10*Δ and the corresponding WT strain were solubilized with digitonin and incubated with affinity matrix coated with GST-Tom22$_{\Delta N}$. Samples were analyzed by SDS-PAGE. Load 1.6%, elution 100%.

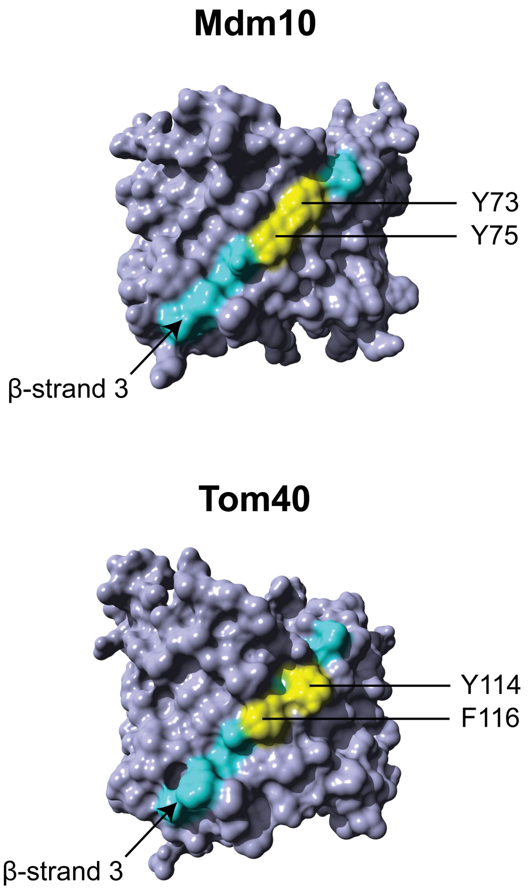

Supplementary Figure 4 | Conservation of SAM binding region of Mdm10 on Tom40. Homology models of the β-barrel domains of Mdm10 (long hydrophilic loops shortened) and Tom40 (ref. 1). β-Strand 3 is indicated in cyan. Mdm10 amino acid residues involved in binding to SAM and the corresponding residues of Tom40 are indicated in yellow.

Figure 1c

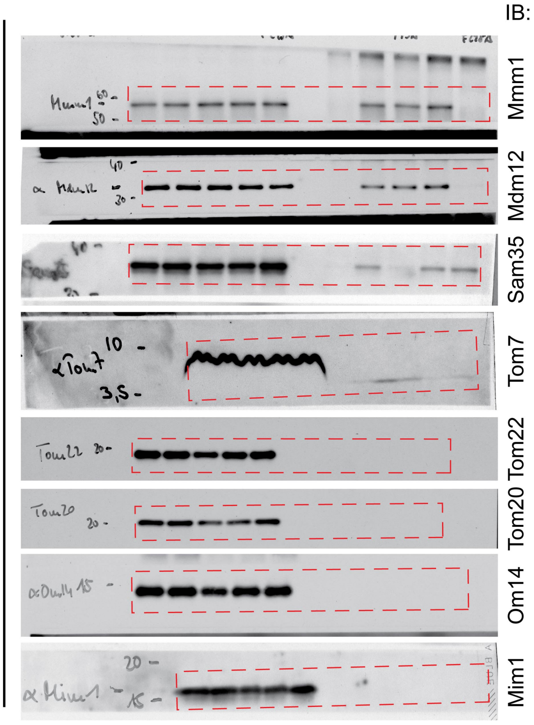

Figure 2a

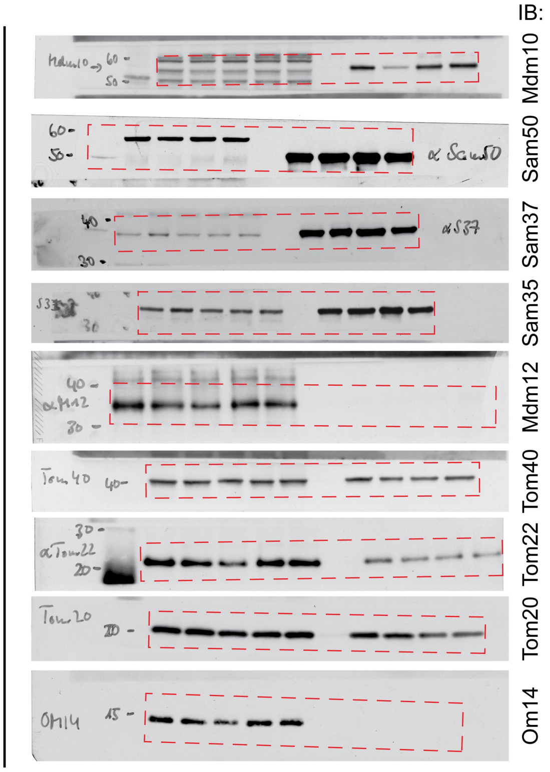

Figure 2b, upper panel

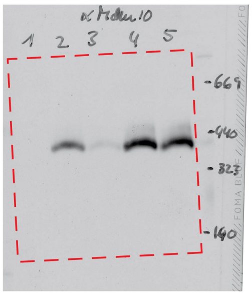

IB: Mdm10

Figure 2b, lower panel

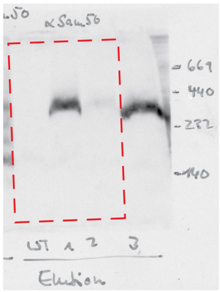

IB: Sam50

Figure 2c

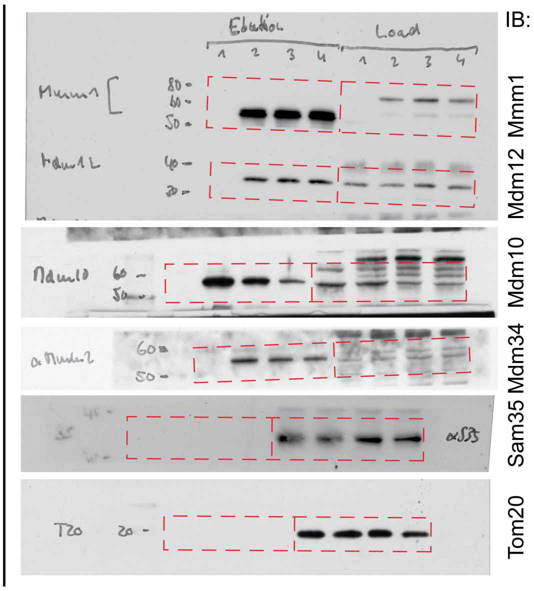

Figure 4a, (pH 10.8) **Figure 4a, (pH 11.5)**

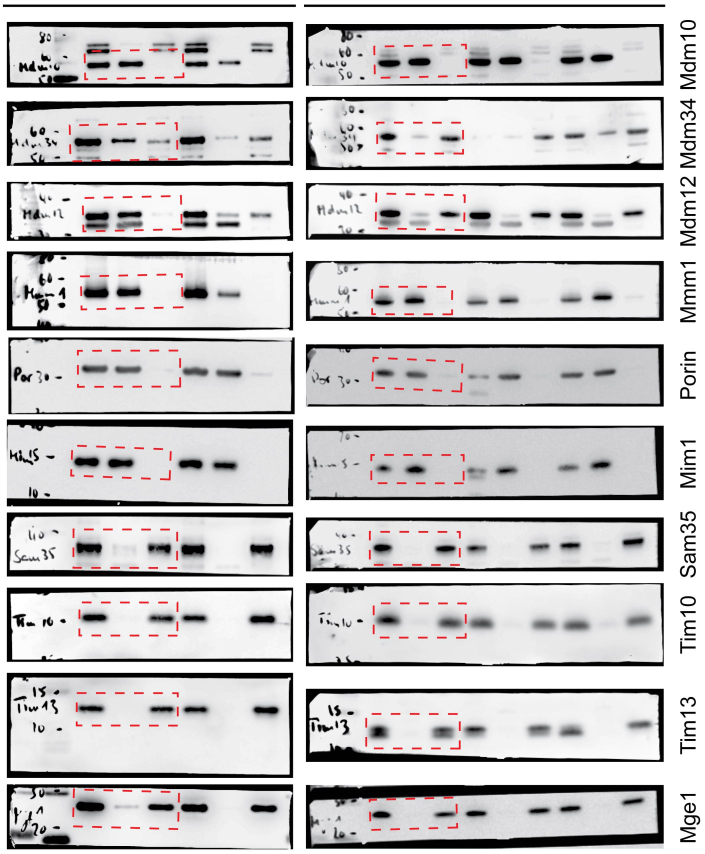

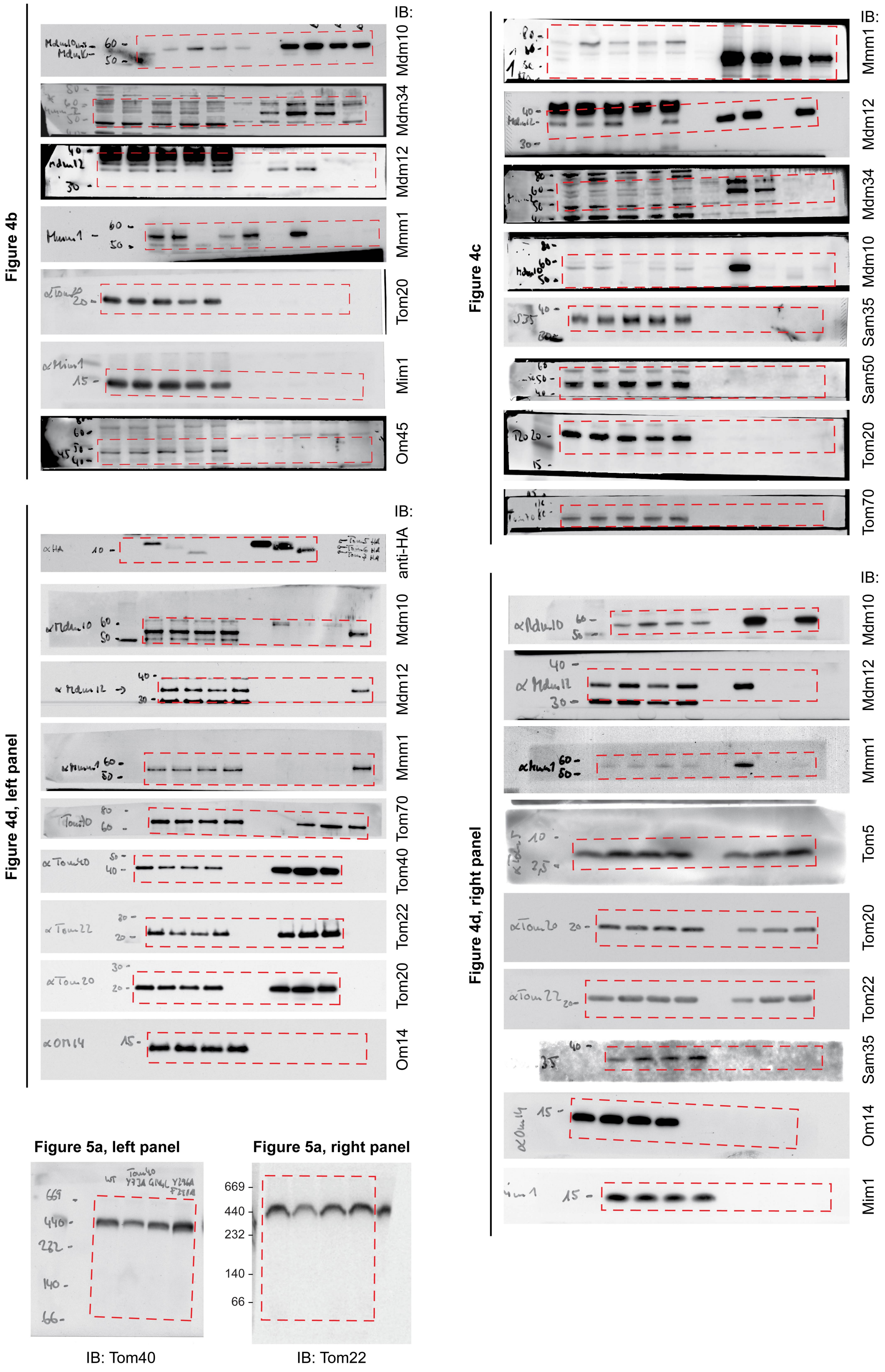

Supplementary Figure 5 I Original scans of key Western blots presented in this paper

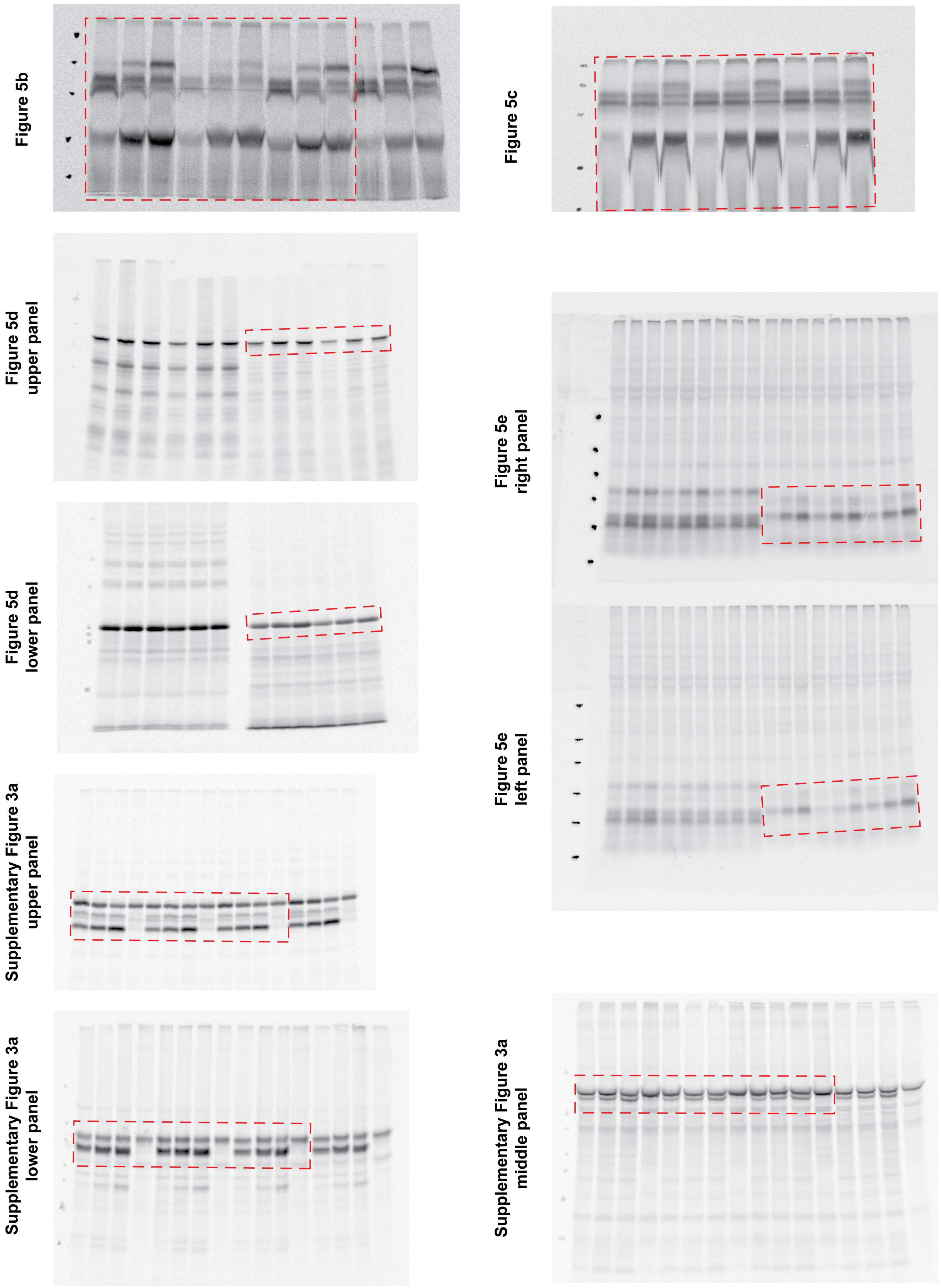

Supplementary Figure 6 I Original scans of key autoradiography gels presented in this paper

Strain	Genetic background	Source	No.
YPH499 (WT)	*MATa ura3-52 lys2-801_amber ade2-101_orchre trp1-Δ63 his3-Δ200 leu2-Δ1*	ref. 2	1501
BY4741 (WT)	*MATa his3Δ1 leu2Δ0 met15Δ0 ura3Δ0*	Euroscarf	1354
Mdm10^{WT} (WT)	YPH499 *mdm10::ADE2* pFL39-Mdm10^{WT}	ref. 1	3402
YPH499 rho^{0} (WT for *mdm10*Δ)	YPH499 rho^{0}	Pfanner Lab	1519
Mdm10$^{Y73.75A}$	YPH499 *mdm10::ADE2* pFL39-Mdm10^{Y73,75A}	this study	4541
Mdm10^{G144L}	YPH499 *mdm10::ADE2* pFL39-Mdm10^{G144L}	this study	4542
Mdm10^{Y296A,F298A}	YPH499 *mdm10::ADE2* pFL39-Mdm10^{Y296A,F298A}	this study	4543
Mdm10^{Y296,301A,F298A}	YPH499 *mdm10::ADE2* pFL39-Mdm10^{Y296,301A,F298A}	this study	4544
$_{ProtA}$Sam50 Mdm10^{WT}	YPH499 *mdm10::ADE2* pFL39-Mdm10^{WT} *sam50::HIS3MX6-NOPI-ProtA-TEV-SAM50*	this study	4553
$_{ProtA}$Sam50 Mdm10^{Y73,75A}	YPH499 *mdm10::ADE2* pFL39-Mdm10^{Y73,75A} *sam50::HIS3MX6-NOPI-ProtA-TEV-SAM50*	this study	4554
$_{ProtA}$Sam50 Mdm10^{G144L}	YPH499 *mdm10::ADE2* pFL39-Mdm10^{G144L} *sam50::HIS3MX6-NOPI-ProtA-TEV-SAM50*	this study	4555
$_{ProtA}$Sam50 Mdm10^{Y296A,F298A}	YPH499 *mdm10::ADE2* pFL39-Mdm10^{Y296A,F298A} *sam50::HIS3MX6-NOPI-ProtA-TEV-SAM50*	this study	4556
$_{ProtA}$Mmm1 Mdm10^{WT}	YPH499 *mdm10::ADE2* pFL39-Mdm10^{WT} *mmm1::HIS3MX6-NOPI-ProtA-TEV-MMM1*	ref. 1	3801
$_{ProtA}$Mmm1 Mdm10^{Y296A,F298A}	YPH499 *mdm10::ADE2* pFL39-Mdm10^{Y296A,F298A} *mmm1::HIS3MX6-NOPI-ProtA-TEV-MMM1*	this study	4547
$_{ProtA}$Mmm1 Mdm10^{Y296,301A,F298A}	YPH499 *mdm10::ADE2* pFL39-Mdm10^{Y296,301A,F298A} *mmm1::HIS3MX6-NOPI-ProtA-TEV-MMM1*	this study	4548
Mdm10$^{WT}{}_{His}$	YPH499 *mdm10::ADE2* pFL39-Mdm10$^{WT}{}_{His10}$	this study	4549
Mdm10$^{Y73,75A}{}_{His}$	YPH499 *mdm10::ADE2* pFL39-Mdm10$^{Y73,75A}{}_{His10}$	this study	4550
$_{HA}$Tom5	YPH499 *tom5::$_{HA}$TOM5*	this study	4052
$_{HA}$Tom6	YPH499 *tom6::$_{HA}$TOM6*	this study	4053
$_{HA}$Tom7	YPH499 *tom7::$_{HA}$TOM7*	this study	4054

$Tom7_{HA}$ $Mdm10^{WT}$	YPH499 *mdm10::ADE2* pFL39-Mdm10^{WT} *tom7::TOM7$_{HA}$-His3MX6*	this study	4568
$Tom7_{HA}$ $Mdm10^{G144L}$	YPH499 *mdm10::ADE2* pFL39-Mdm10^{G144L} *tom7::TOM7$_{HA}$-His3MX6*	this study	4570
$Tom7_{HA}$ $Mdm10^{Y296A,F298A}$	YPH499 *mdm10::ADE2* pFL39-Mdm10^{Y296A,F298A} *tom7::TOM7$_{HA}$-His3MX6*	this study	4571
mdm10Δ (YPH499)	YPH499 *mdm10::kanMX4*	ref. 3	2637
$Mdm10_{His}$	BY4741 *mdm10::MDM10$_{His10}$-HIS3MX6*	this study	4572
$Mdm10_{His}$ *mdm12Δ*	BY4741 *mdm10::MDM10$_{His10}$-HIS3MX6 mdm12::kanMX4*	this study	4573
$Mdm10_{His}$ *mdm34Δ*	BY4741 *mdm10::MDM10$_{His10}$-HIS3MX6 mdm34::kanMX4*	this study	4574
$Mdm10_{His}$ *mmm1Δ*	BY4741 *mdm10::MDM10$_{His10}$-HIS3MX6 mmm1::kanMX4*	this study	4575
$_{ProtA}Mmm1$	BY4741 *mmm1::HIS3MX6-NOPI-ProtA-TEV-MMM1*	this study	4577
$_{ProtA}Mmm1$ *mdm10Δ*	BY4741 *mmm1::HIS3MX6-NOPI-ProtA-TEV-MMM1 mdm10::kanMX4*	this study	4578
$_{ProtA}Mmm1$ *mdm12Δ*	BY4741 *mmm1::HIS3MX6-NOPI-ProtA-TEV-MMM1 mdm12::kanMX4*	this study	4579
$_{ProtA}Mmm1$ *mdm34Δ*	BY4741 *mmm1::HIS3MX6-NOPI-ProtA-TEV-MMM1 mdm34::kanMX4*	this study	4580

Supplementary Table 1 | *Saccharomyces cerevisiae* strains used in this study

Antigen	Dilution	Number/ Company	Secondary Antibody
Mdm10	1:250 TBS + 5% milk	GR1145-3	anti-rabbit
Mdm10	1:40 TBS + 0.5% Tween20	GR1145-3 (affinity purified)	anti-rabbit
Mmm1	1:500 TBS + 5% milk	GR3042-7	anti-rabbit
Mdm12	1:40 TBS + 0.5% Tween20	GR1147-2 (affinity purified)	anti-rabbit
Mdm34	1:200 TBS + 5% milk	GR622-3	anti-rabbit
Mdm34	1:200 TBS + 0.5% Tween20	GR622-5 (affinity purified)	anti-rabbit
Sam50	1:250 TBS + 5% milk	GR312-16	anti-rabbit
Sam50	1:40 TBS + 0.5% Tween20	GR312-17 (affinity purified)	anti-rabbit
Sam35	1:200 TBS + 5% milk	GR551-7	anti-rabbit
Sam37	1:250 TBS + 5% milk	GR161-10	anti-rabbit
Tom7	1:250 TBS + 5% milk	GR230-10	anti-rabbit
Tom5	1:500 TBS + 5% milk	GR3420-5	anti-rabbit
Tom20	1:2000 TBS + 5% milk	GR3225-7	anti-rabbit
Tom22	1:1000 TBS + 5% milk	GR3227-3	anti-rabbit
Tom40	1:500 TBS + 5% milk	GR168-5	anti-rabbit
Tom70	1:500 TBS + 5% milk	GR657-3	anti-rabbit
Mim1	1:500 TBS + 5% milk	GR544-1	anti-rabbit
Om14	1:1000 TBS + 5% milk	GR3041-4	anti-rabbit
Om45	1:500 TBS + 5% milk	GR1311-4	anti-rabbit
Porin	1:1000 TBS + 5% milk	94D	anti-rabbit
Tim10	1:200 TBS + 5% milk	GR2040-4	anti-rabbit
Tim13	1:200 TBS + 5% milk	GR2044-4	anti-rabbit
Atp4	1:250 TBS + 5% milk	GR1970-1	anti-rabbit
Cox1	1:500 TBS + 5 % milk	GR1538-4	anti-rabbit
Cox2	1:500 TBS + 5 % milk	GR1948-4	anti-rabbit
Cox4	1:2000 TBS + 5% milk	GR578-5	anti-rabbit
Mge1	1:250 TBS + 5% milk	GR1837-5	anti-rabbit
Rip1	1:500 TBS + 5 % milk	GR543-5	anti-rabbit
Sdh4	1:500 TBS + 5 % milk	GR1855-5	anti-rabbit
HA	1:1000 TBS + 5% milk	Roche Cat. No.158 3816	anti-mouse

Supplementary Table 2 | Antibodies/antisera used in this study

Supplementary References

1. Flinner, N. *et al.* Mdm10 is an ancient eukaryotic porin co-occurring with the ERMES complex. *Biochim. Biophys. Acta* **1833**, 3314-3325 (2013).
2. Sikorski, R.S. & Hieter, P. A system of shuttle vectors and yeast host strains designed for efficient manipulation of DNA in *Saccharomyces cerevisiae*. *Genetics* **122**, 19–27 (1989).
3. Stojanovski, D., Guiard, B., Kozjak-Pavlovic, V., Pfanner, N. & Meisinger, C. Alternative function for the mitochondrial SAM complex in biogenesis of α-helical TOM proteins. *J. Cell Biol.* **179**, 881–893 (2007).
